# Supplementary material for: Multi-scale time series prediction model based on deep learning and its application
Source: PLoS One. 2025 Jul 10;20(7):e0325474. doi: 10.1371/journal.pone.0325474 (PMC12244652; doi:10.1371/journal.pone.0325474)
Supplement: Attachment — Submitted filename: My_review_PONE-D-24-51425.pdf [file pone.0325474.s001.pdf]

Multi-scale time series prediction model based on deep learning and its application

Review PONE-D-24-51425

The abstract

The abstract introduces several abbreviations, such as MSCNN and MSCALSTM, without adequately defining them or providing sufficient context, which may hinder the reader's understanding.

Introduction and related work

Strengths:

- Clear Context and Motivation: The introduction effectively establishes the importance of addressing traffic congestion in urban areas and frames traffic flow prediction as a cost-effective solution.
- References: It provides a comprehensive overview of traditional and state-of-the-art methods, including statistical, machine learning, and deep learning techniques, with relevant citations.
- Clear Problem Statement: The limitations of existing models, including overfitting, limited scalability, and the challenges of manually designed attention mechanisms, are clearly articulated.

Weaknesses:

1. **Repetition and Redundancy**

- Several points are repeated unnecessarily, such as the advantages of CNN and LSTM, or the improvement in CBAM using multi-scale convolutional layers.
- Example: *"Using convolutions of different scales can enable the module to better model the spatial relationship between features"* appears twice.

2. **Literature Review**

   The overall review of the state-of-the-art methods is relevant and comprehensive, covering traditional statistical models, machine learning techniques, and deep learning approaches. However, I was surprised to see that the combination of wavelet transforms with machine learning models was mentioned only once and that Fourier transform-based approaches combined with nonlinear statistical models were not highlighted. For instance, relevant examples include:

- Wavelet and machine learning combinations for predictive modeling (https://doi.org/10.1016/j.compeleceng.2024.109631).
- Fourier transform applications in air quality forecasting (https://doi.org/10.1016/j.atmosenv.2023.120210).

- Spectral band decomposition with nonlinear models for time series prediction, as in Ouaret et al., *Stochastic Environmental Research and Risk Assessment* (https://doi.org/10.1007/s00477-017-1510-).

Incorporating a discussion on these methods could enrich the literature review by addressing additional perspectives on time series forecasting techniques.

3. Overwhelming Length

- The introduction is dense and too detailed, particularly in the review of related work. While the level of detail may be suitable for the related work section, condensing it in the introduction would improve readability.

4. Focus and Structure

- The introduction tries to cover too many aspects, from statistical methods to recent developments in attention mechanisms, which can dilute the focus.
- The narrative could be more streamlined to emphasize the motivation, limitations, and the novelty of the proposed approach.

5. Clarity in Terminology and Abbreviations

- Abbreviations like ARIMA, MSCALSTM, MSCNN, and MSCBAM are introduced with little explanation, which can confuse readers unfamiliar with the terms.
- Avoid introducing excessive technical terms in the introduction unless necessary.

6. Minor Language Issues

- Some sentences are awkward or unclear. For example: *"Due to the nonlinear characteristics of traffic flow data, overfitting is common in traditional machine learning methods, overfitting is common in traditional machine learning methods..."*

Suggestions for Improvement:

1. **Condense the Literature Review**
   - Focus on the most relevant advancements, such as the transition from statistical methods to deep learning approaches. Move details about individual methods to the related work section.
2. **Streamline and Eliminate Redundancy**
   - Avoid repeating points about multi-scale convolutional layers and the advantages of CNN-LSTM combinations.
3. **Simplify Abbreviation Introductions**
   - Briefly explain MSCALSTM, MSCNN, and MSCBAM when first introduced and use them consistently throughout.
4. **Reorganize for Flow**
   - Start with the problem of traffic congestion and the importance of traffic flow prediction, then outline existing challenges, and conclude with the novelty of the proposed solution.
5. **Refine Language and Remove Errors**
   - Fix repetitive phrases and improve sentence clarity. For example, the sentence about overfitting in machine learning methods could be rewritten as:

"Traditional machine learning methods often suffer from overfitting due to the nonlinear characteristics of traffic flow data, which impacts prediction performance."

6. **Keep the Contributions Section Concise**
   - Avoid repeating the same idea within the contributions, and rephrase for precision.

Method Overview and Experimental Analysis

The methodology of MSCALSTM demonstrates several strengths that make it a compelling contribution. It offers a well-structured breakdown of the model into three main modules—MSCNN, MSCBAM, and LSTM—each tailored to address specific challenges in time series prediction. The multi-scale convolutional approach (MSCNN) is particularly notable for its ability to capture features at varying scales using convolution kernels of sizes 1×1, 3×3, and 5×5, which enhances feature richness, expands the receptive field, and improves prediction accuracy. Additionally, the inclusion of a multi-scale attention mechanism (MSCBAM) adapted from CBAM effectively emphasizes key features and adaptively focuses on important areas, improving both interpretability and performance. The integration of LSTM further strengthens the methodology by addressing the complexity of temporal dependencies, leveraging gating mechanisms to retain long-term information while alleviating the vanishing gradient problem. Moreover, the dimensionality transformations introduced throughout the process ensure compatibility and efficient feature extraction, reflecting careful consideration of the data's structure. Collectively, these elements provide a comprehensive and thoughtful approach to addressing the challenges of spatiotemporal data modeling.

The methodology is clearly structured and demonstrates an understanding of spatiotemporal data modeling. However, the description could benefit from deeper justifications, comparisons with state-of-the-art methods, and discussions on practical applicability and computational efficiency. Including these elements would make the section more robust and impactful.

Points for improvement

A. Clarity and Depth

- The explanation includes detailed equations, but their connection to the overall architecture and contribution to the final prediction could be elaborated more clearly. For instance:
  - How does each module contribute quantitatively to improving prediction accuracy (e.g., ablation studies or comparisons)?
  - Is there evidence that dimensionality transformations improve performance compared to standard input formats?

B. Novelty

- While the methodology combines known components (MSCNN, CBAM, and LSTM), the novelty appears to hinge on using multi-scale convolutions in both MSCNN and MSCBAM.
    - Could the author compare this novelty to existing approaches, particularly hybrid models combining CNNs, attention mechanisms, and LSTMs? For example, how does this model outperform others in literature, such as transformer-based models for spatiotemporal data?

C. Justification of Design Choices

- The rationale for choosing specific kernel sizes (1×1, 3×3, 5×5) and their effect on performance isn't substantiated by experimental results in this section.
    - Why are these kernel sizes optimal for capturing multi-scale features? Could different combinations yield better results?
- While the author claims that MSCBAM enhances interpretability, no specific discussion or metric is provided to demonstrate this interpretability or explain its practical significance.

D. Completeness

- There is no mention of hyperparameter optimization, training details, or computational costs. These aspects are critical for reproducing the results and assessing the method's feasibility.
- The method is described as generalizable, but it would be valuable to discuss its adaptability to datasets or problems beyond traffic flow prediction.

E. Connection to Objectives

- The stated goal is to improve prediction accuracy, robustness, and nonlinear modeling capability. However, the methodology section doesn't sufficiently address robustness or compare the nonlinear modeling capabilities of the proposed approach to existing methods.

F. Missing Comparisons with other decomposition

1. The methodology lacks references to alternative hybrid models and why MSCALSTM is preferable (see previous references in the point literature). For example:
    a. Models combining wavelet transforms and neural networks for temporal feature extraction.
    b. Transformer-based architectures for spatiotemporal data.

To improve the quality of the paper, I suggest the following points :

- **Add Justification for Key Design Choices**: Provide theoretical or experimental justification for the choice of kernel sizes, attention mechanisms, and dimensionality transformations.

- **Enhance Clarity with Visuals**: Include a detailed figure for MSCALSTM's overall structure, with annotations that highlight the interaction between MSCNN, MSCBAM, and LSTM.
- **Incorporate Quantitative Comparisons**: Discuss ablation studies or comparative results showing the improvement brought by multi-scale convolutions and MSCBAM.
- **Expand on Robustness**: Explain how the model handles noise, missing data, or varying input lengths, which are common challenges in time series prediction.
- **Address Computational Costs**: Mention training times, hardware requirements, or the scalability of the approach for large datasets.

Experimental Analysis

- **Absence of Time Series Visualization**: The lack of figures illustrating time series predictions (e.g., comparing actual vs. predicted values across models) diminishes the interpretability and tangible validation of the results. Graphical illustrations would offer a clearer understanding of model performance in capturing patterns and anomalies.
- **Unclear Result Section**: The results are spread across subsections and lack a dedicated "Results" section, making it harder for readers to locate and interpret key findings succinctly. A focused subsection summarizing the comparative performance (e.g., with key takeaways from Tables 1 and 2) would enhance readability.
- **Impact of Computational Complexity**: While MSCALSTM's design is innovative, the computational burden introduced by multi-scale CNNs, CBAM, and attention mechanisms isn't thoroughly analyzed. A brief discussion on scalability for larger datasets would strengthen the evaluation.
- **Limited Baseline Model Interpretability**: While baseline models like MAGRN and AGCRN are described briefly, their key advantages or limitations in relation to MSCALSTM aren't critically compared beyond performance metrics in Table 1.
- **Clarity in Ablation Study Results**: The results of the ablation study (Table 2) are described but lack a detailed quantitative analysis in the text, making it harder to appreciate the precise impact of individual modules.
