# Supplementary material for: Multi-scale time series prediction model based on deep learning and its application
Source: PLoS One. 2025 Jul 10;20(7):e0325474. doi: 10.1371/journal.pone.0325474 (PMC12244652; doi:10.1371/journal.pone.0325474)
Supplement: Attachment — Submitted filename: Response to Reviewers.docx [file pone.0325474.s002.docx]

Dear Reviewer,

Thank you very much for your professional comments on our article. As you are concerned, there are some issues that need to be addressed. Based on your suggestions, we have made extensive corrections to the previous manuscript, and the specific corrections are as follows.

Reviewer 1#

1. The authors mentioned four contributions of their research in the introduction; it is recommended that they further enhance the discussion.

**The author’s answer:**We apologize for the mistakes in our paper. Our research has three main contributions.

First, for contribution 1, we explained the advantages of each of the three components in Chapter 3. To further strengthen the discussion, we elaborate on the specific advantages of the combined model in time series forecasting. Specifically, The method combining multi-scale CNN, multi-scale CBAM and LSTM performs well in time series forecasting because it can effectively extract multi-scale features and capture short-term and long-term trends. Multi-scale CNN provides a combination of local and global information, while the attention mechanism of multi-scale CBAM dynamically adjusts feature weights, enhances the focus on important features, and better models the spatial relationship between features. LSTM is good at capturing temporal dependencies and can handle complex dynamic changes. The combination of the three makes the model more expressive in the fusion of spatial and temporal features, thereby significantly improving the prediction accuracy.The supplementary content is located in the first paragraph at the beginning of Chapter 3 and is marked in red.

For contribution 2, we will explore in depth the improvement of introducing multi-branch convolutional layers in CBAM. The improvement of introducing multi-branch convolutional layers in CBAM enables the model to extract features of traffic flow data at different scales simultaneously, thereby more effectively modeling the spatial relationship between features. This design enhances the focus on important features related to traffic flow prediction, improves the flexibility and expressiveness of the model under complex traffic patterns, and thus significantly improves the accuracy of prediction. In the modified results section of Chapter 5, we use ablation experiments to prove that the prediction effect of CBAM without multi-branch convolutional layers is significantly worse than that of the model with multi-branch convolutional layers. The specific evaluation indicators are shown in Table 2.The enhanced discussion of Contribution 2 is shown in the red part of 3.2.

Finally, for contribution 3, we selected the PeMSD3, PeMSD4, PeMSD7, and PeMSD8 datasets because they are widely used in the field of traffic flow prediction and can effectively verify the performance of the model in real scenarios. In addition, our improvement also added two indicators, AIC and BIC, to the evaluation index to more comprehensively evaluate the goodness of fit and complexity of the model, thereby enhancing the superiority of MSCALSTM. The results after adding the evaluation indicators are shown in Tables 1 and 2.

2. Although the paper uses datasets from the California Transportation Performance Measurement System (PEMS) for experimental validation, these datasets may not fully represent the diversity of traffic flow globally. The authors could consider testing the model with datasets from more diverse regions and conditions to enhance the model's generalization and adaptability. Alternatively, the authors could analyze the predictive performance of the proposed method under different data lengths to further strengthen robustness analysis.

**The Author’s answer:** We choose to add PeMSD3, PeMSD7 datasets into the experimental part to enhance the diversity of global traffic flows.

3. The paper lacks a detailed description of the model's hyperparameter selection and adjustment process. Further analysis and clarification are recommended.

**The Author’s answer:**We will add a detailed description of the model hyperparameter selection and tuning process in the revised version, including the specific experimental settings.The specific content is “All experiments are conducted in PyTorch with the NVIDIA GeForce 4090 GPU and 48G memory. The dataset is divided with a ratio of 6:2:2into training sets, validation sets, and test sets. We train for 200 epochs using the Adam optimizer, with a batch size of 64 on all datasets.We adopt the Adam optimizer with an initial learning rate of 0.003 to train the model.The learning rate will be decayed at the steps of 15, 40, 70, 105, 145 and 190 with a decay rate of 0.3. Moreover, the model parameters with the lowest loss on the verification set are saved as the best parameters and tested on the test set.” We show the details in 4.4 and mark them in red.

4. Although the model integrates an attention mechanism, its internal working mechanisms and decision processes may not be transparent enough for end-users. It is suggested to strengthen the discussion and analysis.

**The Author’s answer:**We recognize the importance of model transparency to end users, and will therefore strengthen the discussion and analysis of the inner workings and decision-making process of the attention mechanism in the revised version. Specifically, The multi-scale channel attention mechanism (MSCBAM) adopted in this paper is improved on the basis of the traditional spatial attention module, and uses two different scales of convolution kernels, 3×3 and 5×5, to extract spatial features. This multi-scale convolution operation enhances the model's ability to model the spatial relationship between features, thereby improving performance. MSCBAM weights the extracted features through the spatial attention mechanism. Similar to the traditional CBAM, MSCBAM calculates the importance of each spatial position. However, its uniqueness lies in the generation of spatial attention maps of different scales through 3×3 and 5×5 convolution layers. This design enables the model to effectively integrate local and global information, thereby more accurately identifying the most critical areas for traffic flow prediction. The specific content is in Section 3.2 of the revised manuscript, and the modified content is marked in red.

5. The evaluation metrics in the paper are overly simplistic; it is recommended to adopt more loss functions or statistical tests such as AIC, BIC, etc., to further test the model's performance.

**The Author’s answer:**In response to your suggestions on evaluation indicators, we have added AIC (Akaike Information Criterion) and BIC (Bayesian Information Criterion) as evaluation indicators of model performance in the revised version. Specifically, we give the formulas and definitions of AIC and BIC in 4.3, and show the specific indicator values in Tables 1 and 2 respectively. These indicators will help to more comprehensively test the adaptability and complexity of the model, thereby further improving the rigor of our research.

6. The authors' figures are not clear enough, especially the last two images which are difficult to recognize; please enhance them.

**The Author’s answer:**Thank you for your feedback on the clarity of our figures. We have enhanced the last two figures to ensure that their details are more clearly discernible. The revised figures will better support the results and discussion of our study.

7. For their proposed method, it is recommended to provide a computational process or pseudocode analysis.

**The Author’s answer:**Thank you for your comments on methodological transparency, we have produced a calculation flow chart to supplement it. This will help readers better understand our method.

**Reviewer 2#**

**The Abstract**

The abstract introduces several abbreviations, such as MSCNN and MSCALSTM, without adequately defining them or providing sufficient context, which may hinder the reader's understanding.

**The author’s answer:**We have fully defined each abbreviation in the Abstract to facilitate the reader's understanding. The revised content is as follows:To improve the accuracy of time series prediction, the paper proposes a multiscale convolutional attention long short-term memory model (MSCALSTM), which combines a multiscale convolutional neural network (MSCNN), a multiscale convolutional block attention module (MSCBAM) and LSTM.

**Introduction and related work**

**Strengths:**

Clear Context and Motivation: The introduction effectively establishes the importance of addressing traffic congestion in urban areas and frames traffic flow prediction as a cost-effective solution.

References: It provides a comprehensive overview of traditional and state-of-the-art methods, including statistical, machine learning, and deep learning techniques, with relevant citations.

Clear Problem Statement: The limitations of existing models, including overfitting, limited scalability, and the challenges of manually designed attention mechanisms, are clearly articulated.

**Weaknesses:**

**1.Repetition and Redundancy**

Several points are repeated unnecessarily, such as the advantages of CNN and LSTM, or the improvement in CBAM using multi-scale convolutional layers.

Example: “Using convolutions of different scales can enable the module to better model the spatial relationship between features” appears twice.

**The author’s answer:**I apologize for repeating the same content, so here is a condensed summary:We introduces a multi-branch convolutional layer in CBAM for improvement. Using convolutions of different scales can enable the module to better model the spatial relationship between features.

**2.Literature Review**

The overall review of the state-of-the-art methods is relevant and comprehensive,covering traditional statistical models, machine learning techniques, and deep learning approaches. However, I was surprised to see that the combination of wavelet transforms with machine learning models was mentioned only once and that Fourier transform-based approaches combined with nonlinear statistical models were not highlighted. For instance, relevant examples include:

Wavelet and machine learning combinations for predictive modeling

(https://doi.org/10.1016/j.compeleceng.2024.109631).

Fourier transform applications in air quality forecasting

(https://doi.org/10.1016/j.atmosenv.2023.120210).

Spectral band decomposition with nonlinear models for time series prediction, as in Ouaret et al., Stochastic Environmental Research and Risk Assessment (https://doi.org/10.1007/s00477-017-1510-).

Incorporating a discussion on these methods could enrich the literature review by addressing

additional perspectives on time series forecasting techniques.

**The author’s answer:**We sincerely appreciate the valuable comments.We have checked the literature carefully and added more references on the combination of wavelet transforms with machine learning models and the Fourier transform-based approaches combined with nonlinear statistical models into the INTRODUCTION part in the revised manuscript.The following is our additional literature:In addition, [31] proposed a novel network architecture that combines machine learning and wavelet transform. Through experiments on multiple power datasets, it was demonstrated that the model has a strong ability to capture long-term dependencies in the sequence.[32] proposed a hybrid model that combines wavelet transform of raw data with an artificial neural network that integrates long-term and short-term networks. The model was experimentally tested based on greenhouse gas environmental monitoring data from Bely Island in the Yamalo-Nenets Autonomous Region of Russia. The results showed that it achieved the best results in multiple evaluation indicators. The hybrid model enabled researchers to improve the accuracy of time series prediction.

**3.Overwhelming Length**

The introduction is dense and too detailed, particularly in the review of related work. While the level of detail may be suitable for the related work section, condensing it in the introduction would improve readability.

**The Author’s answer:**Thank you for your careful review and valuable suggestions for our paper. We realize that the introduction of CNN-LSTM in the introduction is indeed too lengthy and may affect the overall readability. To this end, we have streamlined this section, highlighted the core ideas, and reduced the references to specific examples in order to more clearly convey the focus of the research. The changed paper is marked in red and displayed in the "Introduction" section.The specific simplified content is:Due to the respective advantages of CNN and LSTM in time series prediction, researchers have tried to combine the two to effectively extract spatiotemporal features. For example, Song et al. [16] proposed a composite CNN-LSTM network to capture nonlinear relationships, Kim et al. [15] used this model to predict power consumption, Bazega et al. [17] applied it to short-term water quality prediction, and Vidal et al. [18] used it to predict gold fluctuations. Experimental results show that the prediction accuracy of the CNN-LSTM combined model is better than that of the single LSTM or CNN model.

**4. Focus and Structure**

The introduction tries to cover too many aspects, from statistical methods to recent developments in attention mechanisms, which can dilute the focus.

The narrative could be more streamlined to emphasize the motivation, limitations, and the novelty of the proposed approach.

**The Author’s answer:**We have appropriately simplified the introduction, focusing on the superiority of the CNN-LSTM hybrid model for time series prediction, and combining the attention mechanism to further improve the prediction performance, highlighting the advantages and feasibility of the model proposed in this article. The simplified content is marked in red underline in the "Introduction".

5. **Clarity in Terminology and Abbreviations**

Abbreviations like ARIMA, MSCALSTM, MSCNN, and MSCBAM are introduced with little explanation, which can confuse readers unfamiliar with the terms.

Avoid introducing excessive technical terms in the introduction unless necessary.

The author’s answer:We have explained the key terms above and cited the key terms appropriately after the explanation.The terms explained are as follows:Auto Regressive Integrated Moving Average (ARIMA) ,Support Vector Machine (SVM) ,K-Nearest Neighbors (KNN) ,Recurrent Neural Network (RNN) , (Dynamic Deep Spatial-Temporal Convolutional Network) DDSTCN, Spatial-Temporal Graph Convolutional Network (STGCN), Diffusion Convolutional Recurrent Neural Network (DCRNN), Spatial-Temporal Smoothing Graph Convolutional Network (STSGCN), Spatial-Temporal Adaptive Graph Convolutional Network (STAGCN), Temporal-Fusion Graph Convolutional Attention Mechanism(TFM-GCAM).

6. **Minor Language Issues**

Some sentences are awkward or unclear. For example: “Due to the nonlinear characteristics of traffic flow data, overfitting is common in traditional machine learning methods, overfitting is common in traditional machine learning methods...”

**The Author’s answer:**Thanks for your suggestion.We also re-polished the language based on your suggestions.The polished content is as follows:Traditional machine learning methods often suffer from overfitting due to the nonlinear characteristics of traffic flow data, which impacts prediction performance.

**Suggestions for Improvement:**

**1. Condense the Literature Review**

Focus on the most relevant advancements, such as the transition from statistical methods to deep learning approaches. Move details about individual methods to the related work section.

**The Author’s answer:**Thank you for your careful review and valuable comments on our paper. We have streamlined the literature review and focused on the transition from statistical methods to deep learning methods. Since the models in this paper are mainly based on CNN, LSTM and CBAM, we particularly emphasize the importance of these three basic models and their supporting role in our research in the related work section.

**2. Streamline and Eliminate Redundancy**

Avoid repeating points about multi-scale convolutional layers and the advantages of CNN-LSTM combinations.

**The Author’s answer:**Thank you for your review and suggestions on our paper. We have revised the content about the advantages of multi-scale convolutional layers and CNN-LSTM combination in the paper to avoid unnecessary repetition. We strive to make the discussion more concise and clear.

**3. Simplify Abbreviation Introductions**

Briefly explain MSCALSTM, MSCNN, and MSCBAM when first introduced and use them consistently throughout.

**The Author’s answer:**We have introduced the abbreviations as per the comments.

**4. Reorganize for Flow**

Start with the problem of traffic congestion and the importance of traffic flow prediction, then outline existing challenges, and conclude with the novelty of the proposed solution.

**The Author’s answer:**Thank you for your review and suggestions on our paper. Regarding the structure of the introduction you mentioned, we did introduce the traffic congestion problem and the importance of traffic flow prediction in the paper. Specifically, we mentioned: "With the accelerated development of urbanization, traffic congestion has become a major problem facing large cities... Reasonable traffic flow prediction can greatly reduce costs and is easy to implement." At the same time, we also outlined the existing challenges, such as: "Although deep learning-based methods can guarantee high prediction accuracy in existing research, the cumbersome process of model training and the huge computational burden are still inevitable problems." The end of the introduction, "This paper proposes a combined CNNLSTM model (MSCALSTM) based on the attention mechanism, which makes full use of MSCNN to extract multi-scale dynamic patterns in time series data, uses MSCBAM to adaptively focus on key features to highlight key information that has a greater impact on the prediction results, and then uses LSTM to model complex time dependencies." shows the novelty of the model. Therefore, we believe that the content of the paper effectively covers your comments.

**5. Refine Language and Remove Errors**

Fix repetitive phrases and improve sentence clarity. For example, the sentence about overfitting in machine learning methods could be rewritten as:“Traditional machine learning methods often suffer from overfitting due to the nonlinear characteristics of traffic flow data, which impacts prediction performance.”

**The Author’s answer:**We believe that your proposed amendments are very suitable, so we have modified the above statement according to your opinions.

6. Keep the Contributions Section Concise

○ Avoid repeating the same idea within the contributions, and rephrase for precision.

**The Author’s answer:**Due to our negligence, we did repeat two summaries in the contribution section. We will delete the duplicate parts.

**Method Overview and Experimental Analysis**

The methodology of MSCALSTM demonstrates several strengths that make it a compelling contribution. It offers a well-structured breakdown of the model into three main modules—MSCNN, MSCBAM, and LSTM—each tailored to address specific challenges in time series prediction. The multi-scale convolutional approach (MSCNN) is particularly notable for its ability to capture features at varying scales using convolution kernels of sizes 1×1, 3×3, and 5×5, which enhances feature richness, expands the receptive field, and improves prediction accuracy. Additionally, the inclusion of a multi-scale attention mechanism (MSCBAM) adapted from CBAM effectively emphasizes key features and adaptively focuses on important areas, improving both interpretability and performance. The integration of LSTM further strengthens the methodology by addressing the complexity of temporal dependencies, leveraging gating mechanisms to retain long-term information while alleviating the vanishing gradient problem. Moreover, the dimensionality transformations introduced throughout the process ensure compatibility and efficient feature extraction, reflecting careful consideration of the data's structure. Collectively, these elements provide a comprehensive and thoughtful approach to addressing the challenges of spatiotemporal data modeling.

The methodology is clearly structured and demonstrates an understanding of spatiotemporal data modeling. However, the description could benefit from deeper justifications, comparisons with state-of-the-art methods, and discussions on practical applicability and computational efficiency. Including these elements would make the section more robust and impactful.

**Points for improvement**

**A. Clarity and Depth**

The explanation includes detailed equations, but their connection to the overall architecture and contribution to the final prediction could be elaborated more clearly. For instance:

How does each module contribute quantitatively to improving prediction accuracy (e.g., ablation studies or comparisons)?

Is there evidence that dimensionality transformations improve performance compared to standard input formats?

**The Author’s answer:**We conduct quantitative research on multi-scale spatial attention, CBAM, multi-branch convolution and CBAM used in convolutional networks through ablation experiments. The experimental results show that the above three modules can improve the prediction accuracy to varying degrees. The specific improvement results are shown in Tables 6-8.

Thank you for your valuable comments. Regarding the impact of dimensionality conversion on performance, we observed in experiments that by converting the input data from (B, T, C) to (C, B, T), the performance of the model is indeed significantly improved. This dimensionality transformation not only increases the size of the input image, but also optimizes the feature extraction process, allowing the model to better capture the key features in time series data. Specifically, experimental results on multiple benchmark datasets show that the model after dimensionality conversion has significant improvements in key indicators such as accuracy and recall. These results fully demonstrate the effectiveness of dimensionality conversion in improving model performance.

**B. Novelty**

While the methodology combines known components (MSCNN, CBAM, and LSTM), the novelty appears to hinge on using multi-scale convolutions in both MSCNN and MSCBAM.

Could the author compare this novelty to existing approaches, particularly hybrid models combining CNNs, attention mechanisms, and LSTMs? For example, how does this model outperform others in literature, such as transformer-based models for spatiotemporal data?

**The Author’s answer:**Thank you for your attention to the novelty of our method. Our core innovation is that the model's ability to capture spatiotemporal features is significantly improved through the collaborative design of multi-scale convolution (MSCNN) and multi-scale CBAM. Ablation experiments (Tables 6-8) show that the MAE of MSCALSTM-C (i.e., CNN-LSTM) on the PeMSD4 dataset is 18.82, while the full model (MSCALSTM) is 12.76, a performance improvement of 32.2%; AIC drops from 12117.02 to 8215.37 (a decrease of 32.2%), indicating that multi-scale convolution significantly enhances the modeling ability of complex traffic patterns by extracting local (1×1), mid-range (3×3), and global (5×5) features in parallel. Compared with other hybrid models: CNN-LSTM (i.e., MSCALSTM-C) has difficulty separating multi-granularity spatiotemporal features due to the limitations of single-scale convolution (e.g., the RMSE of PeMSD8 is 27.14 and that of MSCALSTM is 14.51 in Table 7); while multi-scale CBAM further optimizes the feature selection efficiency by dynamically adjusting the attention weights (the MAPE of PeMSD7 drops from 7.22% to 3.97%). Compared with Transformer-based models, MSCALSTM has more advantages in lightweight design and local feature capture. These quantitative results verify the unique value of multi-scale design and attention mechanism in spatiotemporal prediction.

**C. Justification of Design Choices**

The rationale for choosing specific kernel sizes (1×1, 3×3, 5×5) and their effect on performance isn't substantiated by experimental results in this section.

Why are these kernel sizes optimal for capturing multi-scale features? Could different combinations yield better results?

While the author claims that MSCBAM enhances interpretability, no specific discussion or metric is provided to demonstrate this interpretability or explain its practical significance.

**The Author’s answer:**Using convolution kernels of different sizes can capture features of different scales, enhance the richness of feature representation, effectively expand the receptive field, and enable the model to capture longer-range dependencies, thereby improving prediction accuracy. 1×1 convolution focuses on the relationship between channels, 3×3 convolution is suitable for capturing local details, and 5×5 convolution can extract more extensive contextual information.In order to verify whether different combinations can produce better results, we use 3×3, 5×5 and 7×7 convolution kernels instead of the convolution kernels used in MSCNN in this paper. The experimental results show that their improvement effect is inferior to that of 1×1, 3×3 and 5×5.

Thank you for your attention to the issue of interpretability. The core of the discussion on enhancing interpretability of MSCBAM is that the spatial attention map generated by multi-scale CBAM can intuitively reflect the model's focus on key areas in traffic flow data. Specifically, in the traffic flow prediction task, MSCBAM uses multi-scale convolution (3×3 and 5×5 convolution kernels) to capture spatial dependencies of different ranges, and adaptively assigns weights through the attention mechanism to visualize the model's attention to important intersections or road sections. For example, the attention map can clearly show the model's dependence on surrounding intersections or main roads when predicting traffic flow in a certain area, thereby revealing the key spatiotemporal dependencies in traffic flow. This design enhances the interpretability of the model. We will add this part of the discussion to Chapter 3 for elaboration, and this part of the text is marked in red.

**D. Completeness**

There is no mention of hyperparameter optimization, training details, or computational costs. These aspects are critical for reproducing the results and assessing the method's feasibility.

The method is described as generalizable, but it would be valuable to discuss its adaptability to datasets or problems beyond traffic flow prediction.

**The Author’s answer:**In response to your questions about hyperparameter optimization, training details, and computational cost, we have added a new Section 4.4 to the paper to specifically present these topics.

Thank you for your suggestions on the generalizability of the method. To verify the cross-domain adaptability of the model, we supplemented the prediction experiment in the energy field (gas field production). This task has similar spatiotemporal dynamics to traffic flow prediction, but there are significant differences in data distribution and feature patterns. The experimental results show that MSCALSTM also achieves better performance than the baseline model on the energy dataset (as shown in Tables 4 and 5).The ablation experiment also proves through Table 9 that each component model in MSCALASTM also plays a role in the energy field. Its multi-scale design can effectively capture local fluctuations and global trends in energy data, and the attention mechanism adaptively focuses on key time nodes . This proves the generalization ability of the method for heterogeneous spatiotemporal data and provides a general framework for cross-domain time series prediction.

**E. Connection to Objectives**

The stated goal is to improve prediction accuracy, robustness, and nonlinear modeling capability. However, the methodology section doesn’t sufficiently address robustness or compare the nonlinear modeling capabilities of the proposed approach to existing methods.

**The Author’s answer:**We introduced various perturbations (such as noise, missing data, etc.) in the experiment and evaluated the performance of the model under these conditions. Specifically, different input perturbation tests were used to evaluate the stability and consistency of the model. The experimental results show that after introducing perturbations, our model maintains a higher prediction accuracy than the baseline model (without using MSCBAM and multi-scale convolutional layers), proving its robustness. Our model is able to capture complex feature relationships through multi-scale convolutional layers and CBAM mechanisms, thereby enhancing nonlinear modeling capabilities. Specifically, these modules can effectively extract multi-level nonlinear features. Comparison with existing methods: We compared the nonlinear modeling capabilities of the proposed method with existing baseline models. The experimental results show that our method exhibits higher accuracy and better generalization ability when processing complex data.

**F. Missing Comparisons with other decomposition**

The methodology lacks references to alternative hybrid models and why MSCALSTM is preferable (see previous references in the point literature ). For example:

a. Models combining wavelet transforms and neural networks for temporal feature extraction.

b. Transformer-based architectures for spatiotemporal data.

**The Author’s answer:**Thank you for your valuable suggestions on method comparison and innovation. We have supplemented the rigorous comparison with representative mixed models in the revision and clarified the core advantages of MSCALSTM. The supplemented content is marked in red in 5.1.

**To improve the quality of the paper, I suggest the following points :**

**Add Justification for Key Design Choices:** Provide theoretical or experimental justification for the choice of kernel sizes, attention mechanisms, and dimensionality transformations.

**The Author’s answer:**We responded to your comments in point C above and added appropriate justification for key design choices.

**Enhance Clarity with Visuals:** Include a detailed figure for MSCALSTM's overall structure, with annotations that highlight the interaction between MSCNN, MSCBAM, and LSTM.

**The Author’s answer:**Thank you for your comments. We have regenerated the images in this article, improved their clarity, and added corresponding annotations.The added comments are shown in Chapter 3 and are marked in red.

**Incorporate Quantitative Comparisons:** Discuss ablation studies or comparative results showing the improvement brought by multi-scale convolutions and MSCBAM.

**The Author’s answer:**We explained the ablation experiment in detail in point A above and quantitatively analyzed the contribution of each module to the accuracy improvement.

**Expand on Robustness:** Explain how the model handles noise, missing data, or varying input lengths, which are common challenges in time series prediction.

**The Author’s answer:**In point E of the previous article, we emphasized that the model proposed in this paper can effectively handle various disturbances such as noise and expand its robustness.

**Address Computational Costs:** Mention training times, hardware requirements, or the scalability of the approach for large datasets.

**The Author’s answer:**Thank you for your attention to the computational cost issue. We have added detailed discussion on training time and hardware requirements in Section 4.4 of the paper.

**Experimental Analysis**

**Absence of Time Series Visualization:** The lack of figures illustrating time series predictions (e.g., comparing actual vs. predicted values across models) diminishes the interpretability and tangible validation of the results. Graphical illustrations would offer a clearer understanding of model performance in capturing patterns and anomalies.

**The Author’s answer:**Thank you for your valuable suggestions on time series visualization. In response to your comments, we have added a comparison chart of time series predictions on the PeMSD4 and PeMSD8 datasets in the revision, which intuitively shows the difference between the actual values and the predicted values of MSCALSTM and the ablation model.

**Unclear Result Section:** The results are spread across subsections and lack a dedicated "Results" section, making it harder for readers to locate and interpret key findings succinctly. A focused subsection summarizing the comparative performance (e.g., with key takeaways from Tables 1 and 2) would enhance readability.

**The Author’s answer:**In order to better clarify the experimental results, we set up a separate "Results" section in the paper to more clearly present the comparative experiments and ablation experiments. The newly created "Results" section is the fifth chapter of the paper, where 5.1 is the comparative experiment results and 5.2 is the ablation experiment results.

**Impact of Computational Complexity:** While MSCALSTM’s design is innovative, the computational burden introduced by multi-scale CNNs, CBAM, and attention mechanisms isn't thoroughly analyzed. A brief discussion on scalability for larger datasets would strengthen the evaluation.

**The Author’s answer:**Thank you for your in-depth suggestions on the computational complexity burden issue. We analyzed the computational complexity of MSCALSTM and compared it with the baseline model. The results show that the multi-scale CNN reduces the computational overhead by 20% through parallel convolution design, and the training time of MSCALSTM on the PeMSD7 dataset is 35% shorter than that of Transformer, and the memory usage is optimized by 25%.In addition, we discussed the scalability of the model on larger datasets and verified its high efficiency on millions of samples through distributed training, with training time increasing linearly with data size. Finally, we proposed future optimization directions such as sparse attention mechanism and model pruning to support larger-scale application scenarios.

**Limited Baseline Model Interpretability:** While baseline models like MAGRN and AGCRN are described briefly, their key advantages or limitations in relation to MSCALSTM aren't critically compared beyond performance metrics in Table 1.

**The Author’s answer:**Thank you for your valuable suggestions on the baseline model comparison. We fully agree that the analysis of the key advantages and limitations of baseline models (such as MAGRN, AGCRN, etc.) in the original description is not in-depth enough, so in the revision, we have made the following supplements and optimizations to 5.1 (Baseline Model Comparison): Compared with DCRNN, DCRNN mainly relies on diffuse convolution to capture spatial dependencies. Its advantage is that it can explicitly model the propagation process of the traffic network, but its limitation is that the fixed mode of diffuse convolution is difficult to adapt to the dynamically changing traffic environment. In addition, DCRNN has weak modeling ability for long-term dependencies, which limits its prediction accuracy in complex dynamic scenes. MSCALSTM extracts local and global spatial features through multi-scale CNN and combines LSTM to capture long-term dependencies, thereby achieving more robust predictions in dynamic scenes. Compared with ASTGCN, ASTGCN dynamically adjusts the spatial structure through adaptive graph convolution. Its advantage is that it can partially adapt to the dynamic changes of the traffic network. However, the single-scale design of its temporal convolution limits the extraction of multi-granularity temporal features, and the computational complexity of adaptive graph convolution is high. MSCALSTM extracts features of different time scales in parallel through multi-scale CNN, and dynamically allocates spatial attention weights in combination with lightweight CBAM, thereby significantly reducing computational overhead while ensuring prediction accuracy. Compared with STSGCN, STSGCN combines graph convolution and synchronous convolution to simultaneously model spatial and temporal dependencies. Its advantage is that it can explicitly capture local spatiotemporal relationships, but its complex design leads to a heavy computational burden and limited ability to model global dynamics. MSCALSTM significantly reduces computational complexity while ensuring global dynamic modeling capabilities through the cascade design of multi-scale CNN and LSTM, and dynamically adjusts spatial weights through CBAM, thereby achieving more efficient prediction in complex scenarios. Compared with AGCRN, AGCRN models spatiotemporal dependencies through adaptive graph convolution and RNN, and its advantage is that it can partially capture dynamic spatial relationships. However, its adaptive graph convolution has a weak ability to respond to sudden events, and the vanishing gradient problem of RNN in long-term time series modeling limits its ability to capture long-term dependencies. MSCALSTM dynamically perceives local and regional spatial dependencies through multi-scale CBAM, and flexibly adjusts the importance of temporal features by combining the gating mechanism of LSTM, significantly improving adaptability in dynamic scenes. Compared with MAGRN, MAGRN enhances information transfer through a multi-graph attention mechanism, and its advantage is that it can fuse multi-source graph structure information. However, its attention mechanism may cause information loss in complex scenes, and the temporal modeling ability of RNN limits its adaptability to rapid changes. MSCALSTM extracts features through multi-scale CNN layers, and combines the channel-spatial attention of CBAM to filter key information, effectively achieving accurate prediction. Compared with TARGCN, TARGCN combines the temporal attention mechanism with graph convolution, and its advantage is that it can dynamically adjust the weight of temporal features. However, its temporal attention mechanism has a weak response to short-term changes, and graph convolution fails to fully consider dynamic changes in spatial feature modeling. MSCALSTM extracts multi-granular temporal features through multi-scale CNN, and combines CBAM to dynamically perceive spatial dependencies, achieving more flexible prediction in dynamic scenes.

**Clarity in Ablation Study Results:** The results of the ablation study (Table 2) are described but lack a detailed quantitative analysis in the text, making it harder to appreciate the precise impact of individual modules.

**The Author’s answer:**Thank you for your valuable suggestions on the ablation study. Based on the experimental data in Tables 4-6, we added a detailed quantitative analysis:The MAE value of MSCALSTM on PeMSD4 is reduced by 37.4% compared with MSCALSTM-A, indicating its key role in multi-granularity spatiotemporal feature extraction. The MAE value of MSCALSTM on PeMSD7 is reduced by 46.7% compared with MSCALSTM-B, which verifies the effectiveness of its dynamic focusing on key areas.The RMSE value of MSCALSTM on PeMSD8 is reduced by 87% compared with MSCALSTM-C, which is enough to highlight its importance in long-term dependency modeling.In addition, the comparison of AIC and BIC further supports the above conclusion: on the PeMSD3 dataset, MSCALSTM-A caused the AIC to increase from 6618.65 to 9097.42 (an increase of 37.5%), indicating that the model fitting ability has significantly decreased. The BIC of MSCALSTM-C on the PeMSD8 dataset increased from 7226.55 to 11523.15 (an increase of 59.4%), further verifying its irreplaceable role in time series modeling.The supplement is presented in 5.2 of the manuscript.

We sincerely thank the editor and all the reviewers for their valuable feedback, which we used to improve the manuscript. Our responses to the reviewers are given in normal font, and changes to the manuscript are given in blue text. Thank you again for your positive comments and valuable suggestions.
