# Supplementary material for: Multi-scale time series prediction model based on deep learning and its application
Source: PLoS One. 2025 Jul 10;20(7):e0325474. doi: 10.1371/journal.pone.0325474 (PMC12244652; doi:10.1371/journal.pone.0325474)
Supplement: Attachment — Submitted filename: Response_to_Reviewers_auresp_2.docx [file pone.0325474.s003.docx]

kyReviewer 1#

1. **Although the authors have supplemented the description of model training time and hardware requirements, the analysis of the model's scalability and computational efficiency on large-scale datasets remains insufficient. In practical applications, tasks such as traffic flow forecasting and energy forecasting often involve massive amounts of data, where computational efficiency and memory usage are critical factors. It is recommended that the authors further analyze the model's performance on datasets of varying sizes and explore potential optimization strategies, such as distributed computing, model compression, or sparsification, to enhance the model's practicality and scalability.**

**The author’s answer:** To verify the applicability of the model on large-scale data sets, this paper further tests the computational efficiency of MSCALSTM under different data scales. Since traffic flow prediction and energy prediction often involve massive data, in order to better simulate large-scale data scenarios, we expand the original data set through time series generation algorithm and spatial dimension splicing respectively.

Time series generation algorithm: In order to expand the traffic flow data, we use the time series generation algorithm to expand the original data to generate large-scale simulation data. Similarly, we also use this method to expand the energy data set.

Spatial expansion: The detector data of PeMSD3 (358 nodes) and PeMSD7 (228 nodes) are horizontally spliced to construct a 586-node virtual road network (PeMSD3+7) to simulate the complexity of the city-level road network.

In order to verify the performance and scalability of the model in large-scale data scenarios, this paper designed a series of experiments through data expansion and optimization strategies. Through the time series generation algorithm, PeMSD4 (3848 nodes, 50 days of training data) and PeMSD8 (1979 nodes, 62 days of complete time series) were expanded to double the duration, and long sequence synthetic datasets containing 100 days (PeMSD4Large) and 124 days (PeMSD8Large) were constructed. As shown in Table 1, the single CPU training time of the expanded PeMSD4Large increased from 2.8 hours to 5.9 hours, and the memory usage increased from 6.2GB to 12.8GB, but the prediction accuracy MAE only increased slightly from 12.76 to 13.62 (an increase of 6.7%), while the training time of PeMSD8Large increased from 3.2 hours to 6.7 hours, the memory usage increased from 8.1GB to 16.5GB, and the MAE increased from 10.88 to 11.93 (an increase of 9.6%). This shows that the model is significantly robust to long-term dependencies. In the spatial dimension, the detector data of PeMSD3 (358 nodes) and PeMSD7 (228 nodes) are horizontally fused to form a 586-node virtual road network (PeMSD3+7). The video memory usage increases from 6.2GB to 11.8GB, but it can be controlled within 9.5GB through dynamic batch adjustment (batch size is reduced from 64 to 32), and the single sample inference time is still maintained at 19ms (meeting the real-time threshold <50ms). In order to solve the problem of high resource consumption of extended data, a gradient accumulation strategy (steps=4) is further proposed, which reduces video memory usage by 35% and increases training speed by 15% in long sequence training.

**Table 1 Performance comparison on traffic datasets of different sizes**

| **Dataset** | **Data Size** | **Model** | **MAE** | **RMSE** | **MAPE** | **Training Time (h)** | **Memory Usage (GB)** |
| --- | --- | --- | --- | --- | --- | --- | --- |
| **PeMSD4** | 50 days  3848 nodes | MSCALSTM | 12.76 | 17.37 | 5.51% | 2.8 | 6.2 |
| **PeMSD4**  **Large** | 100 days  3848 nodes | MSCALSTM | 13.62 | 18.45 | 6.05% | 5.9 | 12.8 |
| **PeMSD8** | 62 days  1979 nodes | MSCALSTM | 10.88 | 14.51 | 4.40% | 3.2 | 8.1 |
| **PeMSD8**  **Large** | 124 days  1979 nodes | MSCALSTM | 11.93 | 15.89 | 4.82% | 6.7 | 16.5 |
| **PeMSD3** | 358 nodes | MSCALSTM | 10.28 | 15.21 | 6.64% | 2.1 | 5.8 |
| **PeMSD3+7** | 586 nodes | MSCALSTM | 13.15 | 17.92 | 7.20% | 6.5 | 11.8 |

Similarly, to verify the model's long-term time-series adaptability in the energy domain and its practicality in edge computing scenarios, we conducted systematic experiments involving data expansion and model optimization strategies. Firstly, we expanded the existing 30 years of energy data to generate an ultra-long time-series dataset, EnergyLarge (720 months). On this dataset, the prediction error MAE of MSCALSTM increased from 651.37 to 689.45 (a rise of 5.8%), which significantly outperformed the baseline model (HA's increase was 33.2%), indicating that its multi-scale convolution and attention mechanism can effectively capture long-term trend features. Although the training time and memory usage exhibited an approximately linear increase with the sequence length (e.g., memory grew from 13.9GB to 28.4GB), employing a gradient accumulation strategy (steps=4) allowed us to compress it to 10.1GB. Furthermore, targeting edge deployment requirements, we implemented FP16 quantization and parameter sparsification on the LSTM module. This reduced the model size by 40% and decreased the inference latency from 25ms to 18ms (an efficiency improvement of 28%), with a minimal accuracy loss of only 1.1% (MAE 697.21).

**Table 2 Performance comparison on energy datasets of different scales**

| **Dataset** | **Data Size** | **Model** | **MAE** | **RMSE** | **MAPE** | **Training Time(h)** | **Memory Usage (GB)** |
| --- | --- | --- | --- | --- | --- | --- | --- |
| **Energy** | 360 months  （30 years） | MSCALSTM | 651.37 | 668.03 | 13.29% | 3.5 | 7.1 |
| **Energy**  **Large** | 720 months  （60 years） | MSCALSTM | 689.45 | 712.18 | 14.85% | 6.8 | 13.9 |

Likewise, as shown in Table 2, MSCALSTM continued to demonstrate good adaptability in the expanded energy dataset scenario. Even with the extended time series (720 months), its MAE only increased by 5.8%, indicating that the multi-scale convolution and attention mechanism possess a strong ability to capture long-term trends. Furthermore, through quantization and sparsification strategies, the model's inference efficiency on edge devices was improved by 28%, providing feasibility for practical deployment.

**2.The visualization and interpretability of the attention mechanism are inadequate. Despite further discussion on the internal workings of the multi-scale convolutional block attention module (MSCBAM), the authors lack visualization and interpretability analysis of the attention mechanism. A key advantage of attention mechanisms is their ability to reveal the model's focus on input data, thereby enhancing model interpretability. It is suggested that the authors visualize attention weights or attention maps to illustrate the model's focus areas on real data, helping readers better understand how the model identifies and utilizes key features.**

**The author’s answer:** We sincerely thank the reviewers for their insightful suggestions! We fully agree with the value of model interpretability in validating the rationality of our design. In response to your request, we have added a visualization analysis and quantitative discussion of the multi-scale attention mechanism. The specific revisions are as follows:

Taking the multi-scale spatial attention module in MSCBAM as an example. As illustrated in the fig 18, the left panel shows the impact of the 3×3 convolutional kernel on the spatial attention heatmap within MSCBAM. The high-weight regions are concentrated within the 3×3 grid surrounding the target point, indicating that the model prioritizes real-time traffic conditions and can quickly respond to instantaneous changes. The right panel displays the influence of the 5×5 convolutional kernel on the spatial attention heatmap in MSCBAM. This design enables the model to capture long-distance traffic propagation patterns, reflecting long-term trend features, and thus covers a broader area.

**Figure 18. Attention weight heat map**

**3.The authors have provided detailed experimental results in the revised version, but lack statistical significance analysis of the results. When comparing the performance of different models, relying solely on average error metrics (such as MAE and RMSE) may not be sufficient to demonstrate the model's superiority. It is recommended that the authors conduct statistical significance tests (such as t-tests or Wilcoxon tests) to verify whether the improvement in model performance is statistically significant. This will enhance the credibility and persuasiveness of the research findings.**

**The author’s answer:** We appreciate the reviewers for their valuable suggestions. Regarding the statistical significance analysis of the model performance differences, we have added the following work: Based on independent samples t-tests, we systematically examined the MAE of MSCALSTM and all baseline models on the PeMSD3, PeMSD4, PeMSD7, PeMSD8, and Energy datasets. As shown in Table 12 to Table 16, at a significance level of α=0.05, the performance differences between MSCALSTM and each of the comparative models are statistically significant (all p-values are less than 0.001). This analysis further validates that MSCALSTM's superior prediction accuracy is not a random occurrence but arises from the design advantages of its multi-scale feature extraction and dynamic attention mechanism, thereby strengthening the credibility and persuasiveness of the research conclusions.

**Table 12. MAE significance comparison between MSCALSTM and baseline models based on PeMSD3 dataset**

| **Comparison Models** | **t-test statistic** | **P-value** |
| --- | --- | --- |
| **HA** | -41.75 | 2.27e-19 |
| **ARIMA** | -51.15 | 6.03e-21 |
| **LSTM** | -24.89 | 2.15e-15 |
| **STGCN** | -11.82 | 6.48e-10 |
| **DCRNN** | -14.57 | 2.11e-11 |
| **ASTGCN** | -11.38 | 1.18e-09 |
| **STSGCN** | -11.49 | 1.01e-09 |
| **AGCRN** | -9.52 | 1.88e-08 |
| **STG-NCDE** | -9.74 | 1.34e-08 |
| **MAGRN** | -8.47 | 1.08e-07 |
| **TARGCN** | -9.75 | 1.31e-08 |

**Table 13. MAE significance comparison between MSCALSTM and baseline models based on PeMSD4 dataset**

| **Comparison Models** | **t-test statistic** | **P-value** |
| --- | --- | --- |
| **HA** | -52.10 | 4.35e-21 |
| **ARIMA** | -50.82 | 6.78e-21 |
| **LSTM** | -38.99 | 7.67e-19 |
| **STGCN** | -32.03 | 2.52e-17 |
| **DCRNN** | -23.06 | 8.14e-15 |
| **ASTGCN** | -18.15 | 5.09e-13 |
| **STSGCN** | -19.89 | 1.06e-13 |
| **AGCRN** | -16.16 | 3.69e-12 |
| **STG-NCDE** | -11.47 | 1.03e-09 |
| **MAGRN** | -11.62 | 8.43e-10 |
| **TARGCN** | -14.03 | 3.91e-11 |

**Table 14. MAE significance comparison between MSCALSTM and baseline models based on PeMSD7 dataset**

| **Comparison Models** | **t-test statistic** | **P-value** |
| --- | --- | --- |
| **HA** | -63.49 | 1.26e-22 |
| **ARIMA** | -52.87 | 3.34e-21 |
| **LSTM** | -45.88 | 4.33e-20 |
| **STGCN** | -6.93 | 1.78e-06 |
| **DCRNN** | -24.07 | 3.86e-15 |
| **ASTGCN** | -23.80 | 4.69e-15 |
| **STSGCN** | -17.90 | 6.49e-13 |
| **AGCRN** | -16.97 | 1.60e-12 |
| **STG-NCDE** | -13.72 | 5.71e-11 |
| **MAGRN** | -16.19 | 3.57e-12 |
| **TARGCN** | -17.33 | 1.13e-12 |

**Table 15. MAE significance comparison between MSCALSTM and baseline models based on PeMSD8 dataset**

| **Comparison Models** | **t-test statistic** | **P-value** |
| --- | --- | --- |
| **HA** | -52.76 | 3.47e-21 |
| **ARIMA** | -46.50 | 3.32e-20 |
| **LSTM** | -29.82 | 8.91e-17 |
| **STGCN** | -22.78 | 1.01e-14 |
| **DCRNN** | -16.87 | 1.77e-12 |
| **ASTGCN** | -17.75 | 7.48e-13 |
| **STSGCN** | -15.02 | 1.26e-11 |
| **AGCRN** | -11.83 | 6.31e-10 |
| **STG-NCDE** | -10.35 | 5.22e-09 |
| **MAGRN** | -11.75 | 7.08e-10 |
| **TARGCN** | -9.40 | 2.31e-08 |

**Table 16. MAE significance comparison between MSCALSTM and baseline models based on Energy dataset**

| **Comparison Models** | **t-test statistic** | **P-value** |
| --- | --- | --- |
| **HA** | -2519.62 | 2.20e-51 |
| **ARIMA** | -2585.42 | 1.38e-51 |
| **LSTM** | -2498.70 | 2.55e-51 |
| **STGCN** | -1532.39 | 1.69e-47 |
| **DCRNN** | -1746.30 | 1.61e-48 |
| **ASTGCN** | -1068.31 | 1.12e-44 |
| **STSGCN** | -1463.16 | 3.89e-47 |
| **AGCRN** | -972.66 | 6.06e-44 |
| **STG-NCDE** | -727.84 | 1.12e-41 |
| **MAGRN** | -770.39 | 4.03e-42 |
| **TARGCN** | -1008.55 | 3.16e-44 |

As demonstrated in Tables 12 through 16, all the compared p-values are significantly smaller than 0.05, indicating that MSCALSTM's MAE is significantly lower than all the baseline models. The difference is most pronounced when compared to traditional models (HA, ARIMA) and the foundational model (LSTM), where the absolute t-values reach 45 to 63 on PeMSD7, highlighting MSCALSTM's significant advantage in complex spatio-temporal modeling. While the differences compared to some advanced models (such as STGCN and TARGCN) are relatively smaller, with absolute t-values ranging from 6.9 to 22.8, they remain statistically significant. This suggests that the improvements in MSCALSTM's model architecture design consistently lead to better performance.

Reviewer 4#

1. **There are some grammatical errors and sentence structure problems, such as inconsistent punctuation of juxtapositions and loose logical connections in long sentences. It is recommended that the whole text be carefully checked for grammar and language correction to ensure that it is clear and in line with academic writing standards.**

**The author’s answer:** We would like to thank the reviewers for pointing out the grammatical errors and sentence structure issues. We have marked the typical problems and made revisions based on the content of the paper.

(1) Page 1, Abstract: There is no comma before the conjunction "and " in the second sentence of "MSCALSTM model makes full use of the advantages of the above technologies and greatly improves the accuracy and robustness of time series prediction. Extensive experiments are conducted on a dataset from the California Performance Measurement System (PEMS) and the results show that the proposed MSCALSTM model outperforms the state-of-the-art models.", which makes the sentence structure loose.

After: "MSCALSTM model makes full use of the advantages of the above technologies and greatly improves the accuracy and robustness of time series prediction. Extensive experiments are conducted on a dataset from the California Performance Measurement System (PEMS), and the results show that the proposed MSCALSTM model outperforms the state-of-the-art models."

(2) Page 3, Introduction: "In addition, [28] proposed a novel network architecture that combines machine learning and wavelet transform. Through experiments on multiple power datasets, it was demonstrated that the model has a strong ability to capture long-term dependencies in the sequence." The two sentences have weak logical correlation and can be merged into a compound sentence.

After: "In addition, [28] proposed a novel network architecture combining machine learning and wavelet transform, which was validated through experiments on multiple power datasets to demonstrate its strong ability in capturing long-term dependencies."

(3) Page 3, Introduction: There is a missing comma between the sentence "the tedious process of model training and the huge computational burden" inc" Although deep learning-based methods can guarantee high prediction accuracy in existing research, the tedious process of model training and the huge computational burden are still inevitable problems.", which causes ambiguity.

After: "Although deep learning-based methods can guarantee high prediction accuracy in existing research, the tedious process of model training, and the huge computational burden are still inevitable problems."

(4) Page 5: The "channel and spatial dimensions" and "channel attention module and spatial attention module" structures in "CBAM [22] further enhances the connectivity of network features in channel and spatial dimensions by applying channel attention module and spatial attention module sequentially." are repeated and simplified.

After: "CBAM [22] enhances feature connectivity across both channel and spatial dimensions through sequential application of channel and spatial attention modules."

(5) Page 6, Method Overview: "The third module is the LSTM and fully connected layer, which can capture the complex dependencies in the data, perform dimensionality transformation and feature combination, and generate the final prediction results." This sentence contains multiple action descriptions, which are split to improve readability.

After: "The third module comprises LSTM and a fully connected layer. The LSTM captures complex temporal dependencies, while the fully connected layer performs dimensionality transformation and feature combination to generate final predictions."

(6) Page 14, Results: "The experimental results show that MSCALSTM also achieves better performance than the baseline model on the energy dataset (as shown in Tables 4 and 5)." The passive voice "is shown" in this sentence can be replaced with active voice to enhance fluency.

After: "Experimental results demonstrate that MSCALSTM outperforms baseline models on the energy dataset (Tables 4 and 5)."

(7) Page 16, Ablation Study: "The RMSE value of MSCALSTM on PeMSD8 is reduced by 87% compared with MSCALSTM-C, which is enough to highlight its importance in long-term dependency modeling." The word "which" in this sentence is unclear and needs to be supplemented with a logical connective.

After: "The RMSE value of MSCALSTM on PeMSD8 is reduced by 87% compared with MSCALSTM-C. This significant improvement highlights its critical role in long-term dependency modeling."

2. **In the introduction section, the authors mention the limited ability of existing graph convolution methods to capture dynamic spatial relationships, but do not address recent innovative advances in spatio-temporal dependency modelling with graph neural networks. For example, Advanced Engineering Informatics (10.1016/j.aei.2025.103123) proposes an adaptive spatio-temporal modelling framework based on dynamic graph structures, whose multi-scale dynamic pattern extraction can further support the rationality of model design. In addition, the paper emphasizes the role of multi-scale convolution for feature extraction, but no comparative analysis with current cutting-edge methods in the field of feature extraction (e.g., Measurement, 10.1016/j.measurement.2024.115417) is presented, and it is suggested to add relevant discussions to highlight the innovative boundaries of this work.**

**The author’s answer：**We appreciate the reviewers' valuable feedback! We have added recent relevant research on dynamic graph structures in spatio-temporal modeling (such as the DC-DGCN method) to the Introduction section. This addition enhances the completeness of the introduction. Furthermore, we thank the reviewers for their interest in cutting-edge multi-scale feature extraction methods. We have included a comparative analysis with the RTSMFFDE-HKRR method in the Introduction, with a focus on clarifying the novel contributions of our work.

**Supplementary content in the introduction:**

Recent advancements in spatiotemporal modeling frameworks based on dynamic graph structures have demonstrated remarkable advantages in extracting feature correlations. For instance, He et al [28] developed a Dual-Correlation Dynamic Graph Convolutional Network (DC-DGCN) that employs multi-objective optimization to dynamically update graph structures. This approach integrates bidirectional LSTM architecture to enable real-time interaction between physical and digital spaces, effectively capturing multiscale dynamic degradation patterns through coordinated feature learning. In addition, [29] proposed a novel network architecture combining machine learning and wavelet transform, which was validated through experiments on multiple power datasets to demonstrate its strong ability in capturing long-term dependencies.

It is worth noting that multi-scale feature extraction methods have made significant progress in industrial scenarios. For example, the RTSMFFDE-HKRR method proposed by He et al [31] achieves high-precision bearing fault diagnosis in a noisy environment through time-shifted multi-scale fuzzy dispersed entropy and hybrid kernel ridge regression. This method mainly focuses on the local feature decoupling and noise reduction design of mechanical vibration signals. Its multi-scale processing based on entropy theory focuses on the stability of the signal level. Our model realizes the coordinated extraction of road-level micro-features and regional-level macro-features through the parallel design of multi-scale convolution kernels, and dynamically allocates weights in combination with the spatiotemporal attention mechanism. This architecture not only avoids the information loss problem of traditional multi-scale entropy methods in long sequences (such as the coarse-graining process of RTSMFFDE), but also directly models the spatial propagation law of traffic flow through the translation invariance of convolution.
